# Treibhausgasäquivalente und Nutzwasserverbrauch durch dermatologische Produktprobenverpackungen

**DOI:** 10.1007/s00105-024-05392-x

**Published:** 2024-07-23

**Authors:** Dennis Niebel, Carolina Schweig, Esther Luhmann, Susanne Saha

**Affiliations:** 1 Arbeitsgemeinschaft Nachhaltigkeit in der Dermatologie (AGN) e. V., Guntramstr. 8, 79106 Freiburg, Deutschland; 2 Klinik und Poliklinik für Dermatologie, Universitätsklinikum Regensburg, Franz-Josef-Strauß Allee 11, 93053 Regensburg, Deutschland; 3 C.E.Schweig GmbH, Kieler Str. 107, 25474 Bönningsted, Deutschland; 4 Verein demokratischer Pharmazeutinnen und Pharmazeuten, Goldbekufer 36, 22303 Hamburg, Deutschland

**Keywords:** Sampling, Nachhaltigkeit, Kunststoffe, Marketing, Dermatologie, Sampling, Sustainability, Plastic, Marketing, Dermatology

## Abstract

**Hintergrund:**

Unter Sampling versteht man das kostenlose Anbieten kleiner Produktproben. Dabei kann die Verpackung in einem Missverhältnis zum Inhalt stehen, dies führt zu Rohstoffverbrauch und bei mangelnder Recyclingfähigkeit zu Umweltbelastungen.

**Ziel der Arbeit:**

Im vorliegenden Beitrag werden Berechnungen zum Verhältnis zwischen Verpackungs- und Produktgewicht für im Sampling gängige Verpackungsarten (Sachet, Tube, Tiegel) dermatologischer Produktproben dargelegt. Die Zweckmäßigkeit des Samplings wird unter Berücksichtigung umweltrelevanter und betriebswirtschaftlicher Kriterien diskutiert.

**Material und Methoden:**

Insgesamt wurden 43 dermatologische Produktproben unterschiedlicher Hersteller händisch verwogen und klassifiziert. Verpackungen wurden in ihrem strukturellen Aufbau zerlegt. Die anteiligen Gewichte bzw. das Gewicht der Flaschen/Tuben wurden mit Datenbankwerten für den jeweiligen Stoff (Material) bezüglich Treibhausgasäquivalenten (CO2eq) und Brauchwasser verrechnet. Anschließend wurde eine Gesamtsumme für den Impact der jeweiligen Verpackung gebildet. Hierbei wurden nur das Material und dessen Herstellungs- und Verarbeitungsprozess berücksichtigt, weil zu Transport, Gebrauchsnutzen und End-of-Life (EoL) der Verpackungen keine validen Informationen vorlagen.

**Ergebnisse:**

Die kleinste und leichteste Produktprobe (1,24 g) generiert ca. 15 g CO2eq und ca. 700 ml Nutzwasserverbrauch. Die größte und schwerste Produktprobe (37 g) generierte 53 g CO2eq bei 5,78 l Nutzwasserverbrauch. Unter Annahme einer jährlichen Abgabemenge von 10 Mio. Einheiten der hier untersuchten 43 Produktproben entstehen ca. 8000 t CO2eq nur durch die Verpackungen. Weiterhin fallen 880.000.000 l Wasserverbrauch und ca. 2300 t Verpackungsabfall an.

**Diskussion:**

Sampling weist ein ungünstiges Verhältnis zwischen CO2eq/Wasserverbrauch und Nutzen auf, insbesondere im Vergleich zu größeren Verkaufsverpackungen. Produktproben werden jährlich millionenfach in Arztpraxen, Kliniken und Apotheken und insbesondere in der Dermatologie verteilt. Die Praxis des Samplings ist ökologisch und ökonomisch zu hinterfragen.

## Abstract (en)

**Background:**

Sampling refers to the free supply of small product samples. In this process, the packaging can be disproportionate to the contents leading to raw material consumption and, in the case of poor recyclability, environmental pollution.

**Objective:**

In this article, calculations regarding the ratio between packaging and product weight for commonly used types of packaging (sachet, tube, jar) of dermatological product samples are presented. The usefulness of sampling is discussed considering environmental and economic criteria.

**Material and methods:**

A total of 43 dermatological product samples from different manufacturers were manually weighed and classified. Packaging was disassembled into its structural components. The proportional weights or the weight of the bottle/tube body were calculated with database values for the respective material in terms of greenhouse gas equivalents (CO2eq) and freshwater consumption. Subsequently, a total sum for the impact of each packaging was formed. Only the material and manufacturing process were considered because there were no valid data available for transport, utilization, and end of life (EoL) impacts.

**Results:**

The smallest and lightest product sample (1.24 g) generated ca. 15 g CO2eq and approximately 700 ml of freshwater consumption. The largest and heaviest product sample (37 g) generated 53 g CO2eq and 5.78 l of freshwater consumption. Assuming an annual distribution of 10 million units of the 43 product samples examined here, ca. 8000 t of CO2eq are produced by the packaging alone. Additionally, 880,000,000 l of water are used and approximately 2300 t of packaging waste are generated.

**Discussion:**

Sampling shows an unfavorable ratio between CO2eq/water consumption and utility, especially in comparison to larger units of packaging. Millions of product samples are distributed annually in doctor’s practices, hospitals and pharmacies, particularly in dermatology. The practice of sampling should be questioned both ecologically and economically.

Unter Sampling versteht man das kostenlose Anbieten kleiner Produktproben. Diese werden jährlich millionenfach in Arztpraxen, Kliniken und Apotheken – insbesondere in der Dermatologie – verteilt. Dabei fallen große Mengen Verpackungsmaterial an. Herstellung, Verarbeitung, Vertrieb und Entsorgung der verwendeten Materialien führen zur Freisetzung von Treibhausgasen. Die fehlerhafte Entsorgung v. a. von Kunststoffen führt zu zunehmender Umweltbelastung. Die vorliegende Arbeit nähert sich diesem Thema und stellt Berechnungen zu Umwelt- und Klimaauswirkungen des Samplings in der Dermatologie auf. Neben Berechnungen zu Treibhausgasausstoß (gemessen in CO_2_[Kohlendioxid]-Äquivalenten [CO2eq]) und Nutzwasserverbrauch wird das Verhältnis von Aufwand und Kosten gegenüber Marketingeffekten für Unternehmen diskutiert.

## Kunststoffabfälle im Gesundheitswesen und Produktproben

Das Gesundheitssystem in Deutschland ist mit etwa 6,7 % an den nationalen Treibhausgasemissionen beteiligt [12]. Hoher Ressourcenverbrauch spiegelt sich in entsprechendem Abfallaufkommen wider. Die gezielte Reduktion von Kunststoffabfällen ist ein essenzieller Faktor zur Erreichung eines klimaneutralen Gesundheitssektors. Hygienische Anforderungen im medizinischen Bereich und entsprechende gesetzliche Auflagen können allerdings ein Hindernis bei der Abfallminimierung darstellen. Kunststoffabfälle aus dem Gesundheitswesen sind schwer zu recyceln und werden häufig der thermischen Verwertung (Verbrennung) zugeführt. Reduktionsmöglichkeiten bzw. Einsparpotenzial im Kunststoffverbrauch sind im normalen Betriebsablauf von Gesundheitseinrichtungen dennoch möglich. Ganz wesentlich sind 3 Aspekte zu nennen:

1. Verzicht auf Umverpackungen,
2. Reduktion des Kunststoffeinsatzes in Bereichen, in denen dieser nicht zwingend erforderlich ist, und
3. vermehrter Einsatz von recyclingfähigen Kunststoffen sowie ideale Mülltrennung.

Produktproben fallen folglich in Kategorie 2, sie sind für medizinische Behandlungen nicht erforderlich. Viel eher dienen sie als Marketinginstrument, dessen Ziel die Verteilung eines Produkts an Patient:innen und Verbraucher:innen ist, um zum Erwerb der Produkte anzuregen und letztlich den Umsatz zu steigern. Neben dem hohen Aufkommen an Abfall binden die in Praxen, Kliniken und Apotheken eintreffenden Produktproben personelle Ressourcen medizinischer Fachkräfte für die indikationsbezogene Sortierung und Einlagerung. Transportverpackungen sind darüber hinaus teilweise überdimensioniert und enthalten Umverpackungen, Füllmaterial und Empfehlungsblöcke, die häufig gar nicht zum Einsatz kommen. Die Produktproben selbst werden oft in sehr kleinen Einheiten versendet, die Verpackungen bestehen oft aus 2 oder mehr verschiedenen Kunststoffarten für Verschluss und Körper [10]. Nach Ablauf des Haltbarkeitsdatums müssen Produktproben ordnungsgemäß getrennt und entsorgt werden. Es ist jedoch anzunehmen, dass unterschiedliche Verpackungsarten hierbei selten getrennt werden. Auch sind die meisten Produktprobenverpackungen aufgrund der anteiligen Verschmutzung kaum bis nicht recycelbar, für das Sortieren innerhalb des Recyclings ist außerdem eine Mindestgröße erforderlich. Untersuchungen aus dem Vereinigten Königreich zeigen, dass nur 18 % der dermatologischen Produktproben gemäß Herstellerangaben recycelbar sind, während der Rest nicht recycelbar ist oder keine Angaben diesbezüglich vorliegen [15]. Dies kann somit zur Vernichtung von Produkt, Primär‑, Sekundär- und Transportverpackungen beitragen. Da sich die Abfallgebühren in den meisten Kommunen und Städten an der Höhe des Aufkommens orientieren, kann eine Kostensteigerung für die Abnehmer resultieren.

## Umweltaspekte dermatologischer Produktproben und deren Verpackung

Kunststoffe werden aus fossilen Energieträgern wie Erdöl, Erdgas und Kohle hergestellt. Produktion, Lieferketten und Entsorgung von Kunststoffen generieren große Mengen an Treibhausgasen. Neben Kohlendioxid (CO_2_) fallen Methan (CH4) und Lachgas (N_2_O) sowie fluorierte Treibhausgase (F-Gase: wasserstoffhaltige Fluorkohlenwasserstoffe [HFKW], perfluorierte Kohlenwasserstoffe [FKW], Schwefelhexafluorid [SF6], seit 2015 auch Stickstofftrifluorid [NF3]) an. In Deutschland entfielen im Jahr 2020 87,1 % der CO2eq auf CO_2_, 6,5 % auf CH4, 4,6 % auf N_2_O und rund 1,7 % auf F‑Gase [16]. Im Jahr 2015 verursachte die Kunststoffproduktion 4,5 % der weltweit anfallenden CO2eq. Es wird prognostiziert, dass die globale Kunststoffproduktion zwischen 2015 und 2030 um 40 % steigen wird [3]. Vor dem Hintergrund der globalen Erwärmung sind Produktion, Verbrauch und Entsorgung von Kunststoffen kritisch zu hinterfragen [9]: Kunststoffe zerfallen in der Umwelt zu Mikro- und Nanoplastik, die Ökosysteme schädigen können und sich in Nahrungsketten anreichern. Allerdings sind auch andere Verpackungsmaterialien wie Aluminium, Glas und Faserstoffe problematisch für die Umwelt. Für zahlreiche Produktproben (z. B. Sachets) wird Aluminiumfolie als Barriere-gebender Verbundstoffanteil verwendet. Die Gewinnung und Verarbeitung von Aluminium ist sehr energieintensiv. Bei Verbundstoffen mit Aluminium wird nur der Aluminiumanteil zurückgewonnen, der in der Regel unter 30 % liegt, die anderen an der Verbundverpackung beteiligten Kunststoffschichten werden verbrannt [13]. Die Recyclingquote für Aluminium beträgt laut der Deutschen Umwelthilfe (DUH) nur 29,9 % [5]. Auch die Verwendung von Glas als Einwegverpackung generiert relativ viele Treibhausgase. Verantwortlich sind das hohe Gewicht, der damit einhergehende Treibstoffbedarf beim Transport sowie der Energieverbrauch bei der Herstellung. Obwohl Glas im Gegensatz zu Aluminium und Kunststoffen dauerhaft recycelt werden kann, ist der Schmelzvorgang energieintensiv und trägt zu einer schlechten Energiebilanz bei [14]. Im Fall aller Materialien ist jedoch zu berücksichtigen, dass die geringen Produktgrößen beim Sampling einem Recycling im überwiegenden Fall entgegenstehen. Der Wasserverbrauch bei der Produktion von Produktproben stellt ein weiteres erhebliches Problem dar. Weltweit kommt es durch geänderte klimatische Bedingungen zu einer Verknappung von Trinkwasser. Unverhältnismäßigkeit des Wasserverbrauchs steht dem verbindlichen Umweltziel „nachhaltige Nutzung von Wasserressourcen“ der EU-Taxonomie entgegen. Üblicherweise entstehen bei chemischen Herstellungsprozessen Abwässer, die umweltschädliche oder -störende Chemikalien und Schadstoffe enthalten und folglich eine aufwendige Aufbereitung erfordern. Die Aufbereitung von Wasser erzeugt – bei Einsatz fossiler Brennstoffe – ebenfalls Treibhausgase.

## Verpackungsarten dermatologischer Produktproben

Die Praxis des Samplings kann vielgestaltig sein, in dieser Studie wurden konkret 4 Verpackungsklassen identifiziert.

- Sachets

Sachets als Vier- oder Dreikantsiegelbeutel werden meist mit einem Verbundmaterial mit Aluminiumanteil (Folie oder bedampft) gebildet und weisen Füllmengen unterschiedlicher Maßeinheiten auf. Sachets sind – auch wenn sie vollständig entleert werden – aufgrund des Verbundmaterials und ihrer Größe nicht recycelfähig und werden mit dem Restmüll verbrannt. Demgegenüber steht jedoch ein relativ günstiges Verpackungs-Produkt-Verhältnis in Bezug auf das Gewicht.

b) Tuben oder Flaschen aus Kunststoff

Gängige Samples enthalten ein Füllgewicht von 2 ml bis zu 20 ml. Je nach Größe können die Verpackungen zu klein für das Recycling sein. Die schwierige Restentleerung verursacht Probleme, es kommt zu Produktverlust. Im Bereich von 2 ml bis 6 ml weisen Tuben das 1,5- bis 3fache Gewicht des Inhalts auf. Erst ab einem Volumen von 20 ml steht das Verpackungsgewicht in einem angemessenen Verhältnis zum Inhalt.

c) Tuben oder Flaschen aus Kunststoff in Faltschachtel mit Gebrauchsanweisung

Diese Produktmuster entsprechen Kategorie b), allerdings liegen die Tuben oder Flaschen in einem gesonderten Karton. Auffällig bei dieser Art des Samplings ist die Menge an Verpackungsmaterial, es werden Frischfaserstoffe (Kartonagen) mit sehr hohem Umweltimpact verwendet. Die Kartons sind teils überdimensioniert.

d) Einwegglasgebinde (teilweise in Faltschachtel mit Gebrauchsanweisung)

Einwegweißglas als Verpackungsmaterial weist insgesamt den ungünstigsten Klima‑, Wasser- und Ressourcenimpact auf. Um nachhaltiger als eine Kunststoffverpackung mit dem gleichen Volumen zu sein, müssten Verpackungen aus Glas um 40 % leichter sein oder als Mehrwegverpackung verwendet werden [14]. Der letztgenannte Aspekt ist bei Produktproben nicht der Fall.

## Material und Methoden

Insgesamt wurden 43 unterschiedliche Samples, wie in Tab. 1 dargestellt, verwogen. Alle Proben waren mindestens 48 h auf 23 °C 0 % relative Luftfeuchte (rF) klimatisiert. Die Proben wurden mit einer digitalen Feinwaage der TL-Serie von BRIFIT (Shenzhen H-Amier Electronics Technology Co., Ltd., China) mit einem Messbereich von 0,01–50 g verwogen. Die Messungen erfolgen gemäß DIN SPEC 11516:2014 DE. Die zur Prüfung eingesetzten Geräte folgen dem Messmittelmanagement gemäß ISO 9001-312242. Die Probenanzahl der einzelnen Samples umfasste 3 bis 5 Proben, abhängig von der Anzahl der zur Verfügung stehenden Muster. Verwogen und bewertet wurden:

- 16 unterschiedliche Sachet-Typen, Nenninhalt von 0,5 g bis 6 ml in unterschiedlichen Formaten,
- 4 Kunststoffflaschen, Nenninhalt von 15–30 ml,
- 1 Aerosoldose, Nenninhalt von 15 ml,
- 7 Kunststofftuben, Nenninhalt von 2–20 ml,
- 1 Glasflasche (braun) mit Glaspipette und Dosierverschluss, Nenninhalt von 5 ml,
- 13 Flaschen/Tuben mit Faltschachtel oder Promotionkarte, Nenninhalt von 1 g bis 20 ml.

**Tab. 1:** Gesamtübersicht über 43 analysierte Produktproben und deren Verpackung

Sampling Art	Probe	Inhalt	Gewicht (g)	VP entleert (g)	Anteil VP zu Gesamtgewicht (%)	Anteil VP zu Produkt (%)	Material	Produktgewicht (g)	VP Gewicht/100 ml	GHG/Einheit (g)	Wasser/Einheit (l)
*Sachets*	1	0,50 g	1,66	1,24	75	249	Verbund Alu	0,42	62,20	15,98	0,76
	2	2,00 g	3	1,04	35	53	Verbund Alu	1,96	34,53	13,31	0,63
	3	2,00 ml	3,09	1,04	34	51	Verbund Alu	2,05	52,15	13,40	0,64
	4	2,00 ml	3,2	1,33	41	71	Verbund Alu	1,87	66,30	17,03	0,81
	5	2,00 ml	3,4	1,53	45	82	Verbund Alu	1,87	76,70	19,70	0,94
	6	1,50 ml	3,5	2,14	61	158	Verbund Alu	1,36	142,73	27,50	1,31
	7	2,00 ml	3,62	1,59	44	79	Verbund Alu	2,03	79,60	20,45	0,97
	8	3,00 ml	4,56	1,31	29	40	Verbund Alu	3,25	65,60	16,85	0,80
	9	4,00 ml	5,58	1,66	30	42	Verbund Alu	3,92	41,48	21,31	1,01
	10	6,00 ml	7,5	2,58	34	52	KS	4,92	42,95	33,10	1,57
	11	5,00 ml	6,86	1,61	23	31	Verbund Alu	5,25	32,22	20,69	0,98
	12	5,00 ml	6,44	1,15	18	22	Verbund Alu	5,29	23,02	14,78	0,70
	13	2,00 ml	3,19	1,21	38	61	Verbund Alu	1,98	60,50	15,54	0,74
	14	1,00 ml	2,2	1,29	59	143	Verbund Alu	0,91	129,40	16,62	0,79
	15	6,00 ml	8,9	2,64	30	165	Verbund Alu	6,26	44,05	33,95	1,61
	16	5,00 ml	7,036	1,98	28	157	Verbund Alu	5,05	39,64	25,46	1,21
*Flaschen*	17	15,00 ml	19,3	3,82	20	25	KS	15,48	25,48	11,77	1,57
	18	15,00 ml	21,8	6,59	30	43	KS	15,21	43,96	20,31	2,70
	19	15,00 ml	23,6	10,49	44	80	KS	13,12	69,90	32,29	4,30
	20	15,00 ml	25	Details in Fußnote	Nicht entleerbar/trennbar	–	Aludose mit KS-Deckel	–	–	–	–
	21	30,00 ml	41,7	9,49	23	20	KS	32,21	31,63	29,23	3,89
*Tube*	22	3 ml/2 g	5,25	2,42	46	85	KS	2,83	80,57	7,44	0,99
	23	2,00 ml	5,47	2,11	38	63	KS	3,37	105,25	6,48	0,86
	24	2,00 ml	5,99	3,32	55	125	KS	2,67	166,20	10,24	1,36
	25	5,00 ml	7,6	3,59	47	90	KS	4,01	14,36	11,06	1,47
	26	10,00 ml	13,3	3,96	30	38	KS	9,35	39,55	12,18	1,62
	27	20,00 ml	25,3	4,70	19	17	KS	20,61	23,48	14,46	1,92
	28	20,00 ml	25,7	4,78	19	17	KS	20,92	23,91	14,73	1,96
	29	5,00 ml	41,6	Details in Fußnote	89	738	Glas	44,79	738,00	52,63	5,78
*Tuben mit Faltschachteln*	30	2 ml/1 g	4,1	3,07	83	487	KS	1,03	243,50	12,82	2
	31	5,00 ml	7	3,63	70	229	KS	3,37	154,60	19,08	3,22
	32	5,00 ml	6,2	2,70	62	933	KS	3,50	112,00	14,05	2,35
	33	5,00 ml	7,34	3,70	63	79	KS	3,64	122,36	15,95	2,52
	34	7,00 ml	10,1	4,24	56	88	KS	5,87	104,79	17,94	2,76
	35	5,00 ml	9,89	4,10	55	87	KS	2,79	142,00	18,21	2,92
	35	7,50 ml	10,1	3,42	48	93	Aluminiumtube + KS-Deckel	3,88	82,99	15,81	2,57
	37	5,00 ml	7,2	4,20	70	140	KS	3,00	140,40	18,14	2,88
	38	2,00 ml	5,9	2,03	56	52	KS	3,87	241,50	11,86	2,04
	39	7,50 ml	9,4	6,12	77	187	KS	3,28	142,93	27,58	4,43
	40	2,00 ml	3,2	2,10	85	190	KS	1,11	302,25	14,35	2,56
	41	15,00 ml	20,5	8,04	50	65	KS	12,46	82,93	33,7	5,21
	42	20,00 ml	28,1	7,15	40	20	KS	13,95	70,75	35,33	5,86
	43	5,00 ml	9,3	3,70	54	118	KS	5,60	132,28	16,91	2,73

*VP* Verpackung, *KS* Kunststoff, *GHG* Treibhausgas (entspricht CO_2_ [Kohlendioxid])

Details zu Probe 20: Aluminium + Ventil + Kappe

Details zu Probe 30: Behälter (Glas): 32,37 g; Glasrohr: 2,19 g; Schraubdeckel: 1,18 g; Silikon: 1,07 g

Es wurden 42 Proben (Sachets, Flaschen und Tuben) jeweils 2‑mal verwogen – mit Inhalt und anschließend nach Entleerung mittels Rakel, der bei Tuben und Sachets eine ca. 97- bis 98 %ige Restentleerung sicherstellt. Bei Flaschen ist die komplette Restentleerung schwerer messbar; eine Probe mit Aluminiumverpackung (Probe 20) konnte nicht restentleert werden, daher entfiel die Leergewichtbestimmung. Bei Flaschen und Tuben in Faltschachteln wurden die Flaschen und Tuben mit Inhalt verwogen. Alle Faserstoffanteile wie Faltschachteln, Gebrauchsanweisungen und Infokarten wurden separat verwogen, um nicht zu viel Varianz durch die Addition der Packmittel einzubringen. Bei den Sachets wurden Varianzen von ± 5–8 % und bei Flaschen und Tuben wurden Varianzen von ± 8–15 % vermerkt.

Zur Berechnung der CO2eq und des Brauchwassers der Sampling-Verpackungen wurden die aktuellen Daten der Packaging Life Cycle Assessment(LCA)-Software von PIQET (Life Cycle Strategies Pty. Ltd., Collingwood, Australia) im Mai 2024 verwendet (https://piqet.com). PIQET ist ein LCA-Softwaretool, mit dem die Umweltauswirkungen und Ressourcenverbrauchsprofile verschiedener Verpackungsoptionen nach ISO 14040 berechnet werden können. Das Tool bezieht seine Daten von Ecoinvent (Zürich, Schweiz, https://ecoinvent.org), einer zentralen Datenbank, die eine Vielzahl von Sektoren abdeckt. Diese enthält mehr als 20.000 Datensätze zu errechneten Werten in Bezug auf Umweltbelastungen (u. a. CO2eq und Brauchwasser) unterschiedlicher Materialien (relativ zu ihrem Gewicht) und entsprechenden Verarbeitungsprozessen, um menschliche Aktivitäten zu modellieren. Es handelt sich um Mittelwerte bezogen auf Ursprungs- und Verarbeitungsländer. Durch Verwendung dieser Werte und die Verrechnung mit den ermittelten Gewichten der Muster, kombiniert mit den entsprechenden Materialien und Prozessen, können die entsprechenden CO2eq-Werte und Brauchwasserbedarfe für die Rohstoffe und Verarbeitung ermittelt werden. Dieses Vorgehen entspricht der Erstellung eines partiellen *Carbon Footprints* nach DIN EN ISO 14067. Die 43 untersuchten Samplings wurden hierzu zunächst in ihre verschiedenen Verpackungsbestandteile wie Kunststoffe, Aluminium oder Papier zerlegt. Anschließend wurde für jeden Werkstoff einschließlich Kunststoffart, Folienverwendung und Haftkleber der jeweilige Anteil an der Verpackung berechnet. Die Berechnung des jeweiligen Gewichtsanteils erfolgte bei Folien und Papieren pro Quadratmeter derselben Gewichtsklasse oder bei Flaschen pro Stück. Die anteiligen Gewichte der Samplings wurden anschließend mit den Datenbankwerten der LCA-Software für die jeweiligen identifizierten Werkstoffe oder Prozesse verrechnet, um eine Gesamtsumme zu bilden. Alle Berechnungen berücksichtigen den Material- und Herstellungsprozess, jedoch nicht die Lieferketten, den Gebrauchsnutzen und die End-of-Life (EoL), da hierzu keine Aussagen getroffen werden können.

## Ergebnisse

**Abb. 1:**
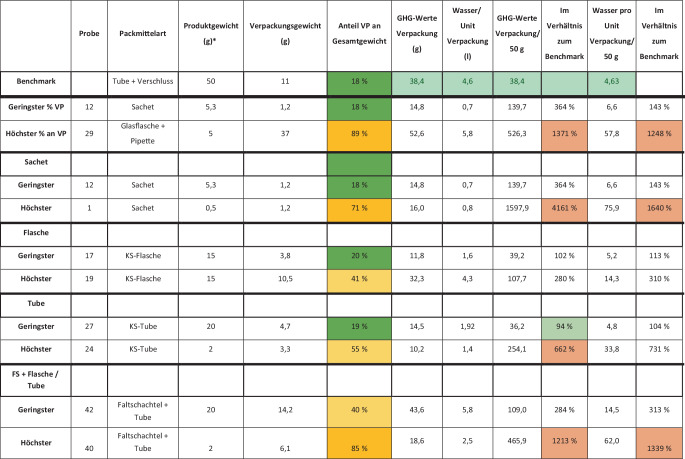
Kurzübersicht der analysierten Proben in Bezug auf Sampling-Art, Bewertung nach Anteil des Verpackungsgewichtes zum Gesamtgewicht und Umweltimpact (CO2eq [Treibhausgasäquivalent], Brauchwasser). Alle Verpackungen wurden im Verhältnis zu einer branchenüblichen (im Vergleich zu anderen Branchen eher minimalisierten) Kunststoffverpackung von 50 ml gesetzt (Benchmark). *Sternchen*: Produktgewicht mit einer theoretischen spezifischen Dichte von 1 g/cm^3^; *VP* Verpackung, *KS* Kunststoff, *GHG* Treibhausgas (entspricht CO_2_ [Kohlendioxid]), *FS* Faltschachtel

Die kleinste und leichteste Produktprobenverpackung (1,24 g) generiert ca. 15 g CO2eq und ca. 700 ml Nutzwasserverbrauch, die größte und schwerste (37 g) generiert 53 g CO2eq bei 5,78 l Nutzwasserverbrauch (Tab. 1 und Abb. 1). Insgesamt wurden durch die Verpackungen der analysierten 43 Proben 0,820 kg CO2eq, 87,96 l Nutzwasserverbrauch und 0,224 kg Verpackungsmüll generiert (Tab. 1). Unter Annahme einer jährlichen Abgabemenge von 10 Mio. Einheiten der hier untersuchten 43 Produktproben entstehen durch die Verpackungen ca. 8000 t CO2eq. Dies entspricht ca. 26-mal der Flugstrecke Hamburg – München. Weiterhin fallen 880.000.000 l Brauchwasser und ca. 2300 t Verpackungsabfall an. Bei Probe 29 wurde eine Glasverpackung gewählt. Hier wurde ein Glastiegel mit einem Kunststoffdeckel, einer Glaspipette und einem Elastomer-Balg kombiniert. Insgesamt wiegt das Packmittel knapp 37 g. Mit diesen 37 g Rohstoff werden 5 ml = 5 g Produkt verpackt. Bei einer Sachet-Verpackung mit gleichem Fassungsvolumen müsste man nur 1,2 g Rohstoff für die Verpackung aufwenden (vgl. Probe Nr. 12). Bei einer Tube inklusive Verschluss müssten 3,6 g Rohstoff eingesetzt werden (vgl. Probe 25). Bei einer exemplarischen Kunststoffflasche inklusive Verschluss läge man bei ca. 4,2 g (vgl. https://flaschen-handel.eu). Weiterhin fällt Probe 20 auf: Sampling einer Aluminiumdose mit Dosierventil und Schutzkappe. Bei Aluminium-Recyclat mit der Gefahr leichter Legierungsverunreinigungen würde bei kleinen Gebinden die Tendenz zu Stressbruch an den Bodenkanten steigen. Verpackungen in dieser Größe werden daher erfahrungsgemäß nicht aus Recyclat-Aluminium hergestellt. Berechnet man das CO2eq einer solchen Produktverpackung aus Aluminium pro Jahr, wenn maximal ein Sampling pro Mailing mit je 20 Proben an 3529 dermatologische Praxen in Deutschland erfolgen würde, entstünden 29 t CO2eq. Zum Vergleich: Eine Produktprobenverpackung aus Kunststoff inklusive Verschluss läge bei ca. einem Sechstel dieses Wertes.

**Abb. 2:**
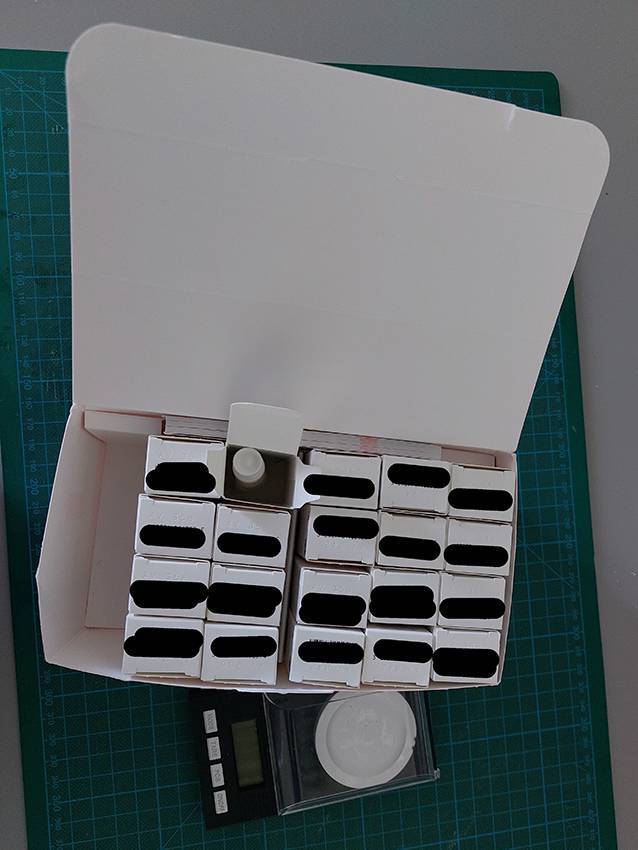
Branchenüblicher Displayaufsteller inklusive Begleitmaterial aus Papier. (Mit freundl. Genehmigung, ©C. Schweig, alle Rechte vorbehalten)

**Abb. 3:**
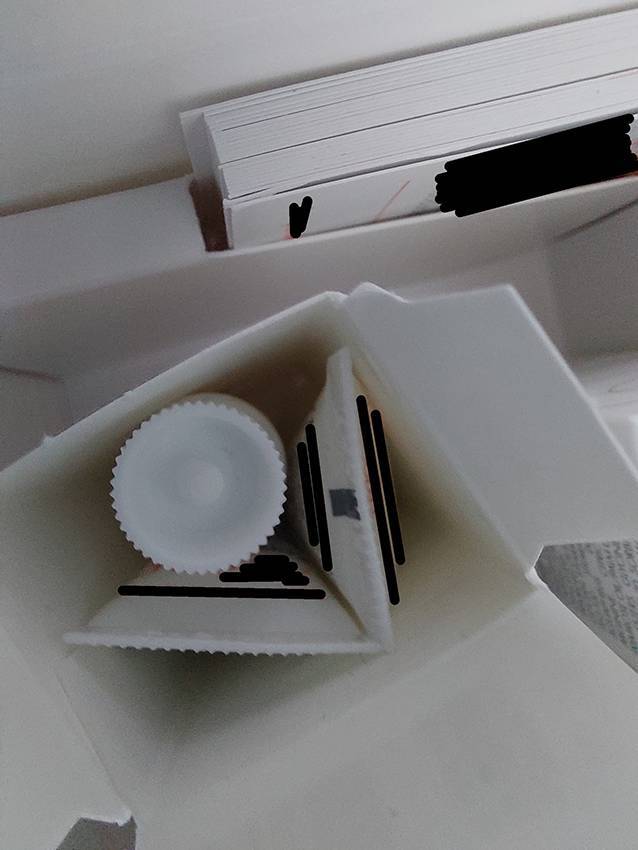
Detailaufnahme einer Faltschachtel. Das Bild zeigt, dass in die vorgegebene Faltschachtel, in der aktuell eine Tube verpackt ist, 3 Tuben passen würden. (Mit freundl. Genehmigung, ©C. Schweig, alle Rechte vorbehalten)

Die Grundlage von Nachhaltigkeit sind Effektivität und Effizienz. Effektivität ist die exakte Erfüllung einer Anforderung, also im Fall von Samplings die Vermeidung von Über- und Unterverpackung. Effizienz ist der minimale Einsatz bei maximalem Nutzen. Um die eingesetzten Verpackungen zu bewerten, wird daher eine Referenz (Benchmark) benötigt. Als Benchmark wurde eine Verpackung für eine Creme in einer handelsüblichen High-Density-Polyethylene(HDPE)-Tube mit einer durchschnittlichen Applikationsmöglichkeit und einem relativ kleinen Schraubdeckel gesetzt (Abb. 1). Für den Produktinhalt wurde ein Referenzwert von 50 ml festgelegt. Bei einer spezifischen Produktdichte von etwa 1 g/cm^3^ aus HDPE entspricht das einem Produktgewicht von 50 g. Diese 50 g Produkt werden mit einem durchschnittlichen Ressourcenimpact (Tube/Verschluss) von ca. 11 g (gemäß Datenblatt: https://linhardt.com/download) verpackt, was zu einer Ratio von ca. 18 % des Verpackungsgewichtes zum Gesamtgewicht führt. Bei genauerer Betrachtung sind mit dieser Benchmark 38,40 g CO2eq und 4,63 l Brauchwasser assoziiert. Die hier vorliegenden Ergebnisse zeigen, dass die mit Sampling-Verpackung verbundenen CO2eq bis zu 4000 % und der Brauchwasserbedarf bis zu 1600 % oberhalb der Referenzverpackung (Benchmark) liegen. Am Beispiel eines branchenüblichen Displayaufstellers für Samplings mit 24 umverpackten Produktproben lässt sich der zusätzliche Umweltimpact der Kartonagen gut darstellen (Abb. 2). Der Displayaufsteller besteht aus Chromokarton, einer der hochwertigsten Kartonarten in der Verpackungsindustrie. Er wiegt 104 g inklusive Innenleben und Infokarten. Jede Faltschachtel wiegt ca. 3 g. Wenn jede der 3529 dermatologischen Praxen 1‑mal im Jahr 2 solche Displaykartons erhält, summiert sich das eingesetzte Kartonmaterial auf 1,23 t pro Jahr mit einem CO2eq von 2,84 t und einem Wasserverbrauch von 693.115,2 l. Bezieht man die Tube als Primärpackmittel mit ein, erhöht das die Treibhausgasemissionen für dieses Sampling um 2,4 t CO2eq, und die Brauchwasserwerte erhöhen sich auf 876.226,9 l. Dieser Verbrauch steht im Vergleich zu ca. 0,6 t Produktinhalt. Interessanterweise würden in den Karton 3 Tuben passen (Abb. 3) – in den Faltschachteln befindet sich aber nur eine Tube. Dies verdeutlicht die überdimensionierte Umverpackung.

## Diskussion

Die hier vorliegende Untersuchung zeigt belastbare Determinanten für den erheblichen ökologischen Impact durch Sampling in der Dermatologie auf. Der Verpackungsaufwand ist, gemessen an der Größe der Produktproben, unverhältnismäßig hoch. Die Entsorgung und das Recycling von Produktproben gestalten sich aufgrund ihrer geringen Größe und der oft komplexen Materialzusammensetzung schwierig und werden wahrscheinlich in vielen Fällen nicht sachgemäß durchgeführt. Dadurch gehen Wertstoffe für das Recycling verloren und werden stattdessen mit dem Restmüll thermisch verwertet. Dies führt nicht nur zu einer Verschwendung wertvoller Ressourcen, sondern auch zu erhöhten Umweltauswirkungen durch die Emission von Treibhausgasen und Schadstoffen. In diesem Zusammenhang ist anzunehmen, dass auch ein Teil der Proben durch Ausflüge und Reisen in die Umwelt gelangt oder in Ländern entsorgt wird, die kein geeignetes Abfallentsorgungs- oder Recyclingsystem besitzen.

In der modernen Unternehmensführung wird die Effizienz aller Marketingmaßnahmen gemessen, unabhängig davon, ob es sich um Print‑/Onlineanzeigen, Veranstaltungen oder eben die Verteilung von Samplings handelt [2]. Dabei werden alle für die Maßnahme notwendigen Faktoren wie Kosten, Arbeitsaufkommen, Investment oder Umweltimpact erfasst und einem definierten Ziel, z. B. einer Erstkauf- oder Wiederkaufrate oder Markenbekanntheit, gegenübergestellt. Zur Messung der Effizienz bei Marketingmaßnahmen – wie dem Sampling – existieren verschiedene Metriken, sog. Key Performance Indicators [1]. Von diesen seien 5 zentrale Indikatoren aufgeführt: Marketingbeitrag zum Umsatz, Pipeline-Wachstum und -Beschleunigung, Conversion-Rate, Kosten pro Lead und Markenbekanntheit. Diese sind für dermatologische Samples nur bedingt anwendbar, da die Nachverfolgung nach Eintreffen der Sendungen in Praxen und Apotheken nicht möglich ist. So lässt sich kein messbarer Unternehmensumsatz auf die Verteilung von Samples zurückführen (Marketingbeitrag zum Umsatz). Auch lässt sich kaum oder keine gezielte und gerichtete Kommunikation mit Verbraucher:innen durchführen (Pipeline-Wachstum und -Beschleunigung). Es ist de facto nicht messbar, ob ein Sample zum Kauf eines Produktes geführt hat (Conversion-Raten), und es ist nicht anzunehmen, dass ein günstiges Kosten-Nutzen-Verhältnis erreicht werden kann, da nicht sicher ist, ob die Proben tatsächlich zu potenziellen Kunden gelangen (Kosten pro Lead). Trotzdem wird Sampling bei den meisten Unternehmen noch als effektives Marketinginstrument wahrgenommen. Der *Mere-Exposure-Effekt* wird durch die kontinuierliche Abgabe großer Mengen von Produktproben an Praxen, Kliniken und Apotheken eingesetzt [8]. Bei diesem Effekt spielen die Menge und die Häufigkeit der Probenverteilungsintervalle eine umsatzrelevante Rolle, um sich gegen Mitbewerber durchzusetzen. Auch als „Branding“ bezeichnet, beruht dieses Instrument auf der stetigen Darbietung der immer gleichen Logos und Produkte und verstärkt so deren individuelle positive Wahrnehmung und damit ihre Markenbekanntheit. Intensiviert wird dieser Effekt über das durch die Sozialwissenschaften definierte universelle *Prinzip der Reziprozität*: Die kostenlose Abgabe von Produktproben kann bei Ärzt:innen den Wunsch auslösen, den Gefallen durch Empfehlung oder im Falle von Patient:innen oder Verbraucher:innen durch Kauf zu erwidern. Exakte Leistungskennzahlen von Sampling-Aktionen können jedoch, wie beschrieben, nicht ermittelt werden, weil sich die Verfolgung von der Produktabgabe bis zum Endverbraucher nicht erfassen lässt. Die Verteilung der Samplings erfolgt damit deutlich überdimensioniert und wenig ressourceneffizient.

Die Verteilung der Samplings erfolgt deutlich überdimensioniert und wenig ressourceneffizient

Für Praxen, Kliniken und Apotheken bedeutet die Beanspruchung der Arbeitszeit von Mitarbeiter:innen einen Verlust an Ressourcen, die der Betreuung von Patient:innen oder der Organisation vorbehalten sein sollte. Die 2022 durch das Bundesgesundheitsministerium geförderte Studie der Stiftung Viamedica zeigt, dass in Praxen, Kliniken und Apotheken der hohe Kosten- und Zeitaufwand sowie der Personalmangel wichtige Gründe sind, weshalb Nachhaltigkeitsmaßnahmen schwer umsetzbar sind [17]. Angesichts der Vielzahl an Maßnahmen, die im Gesundheitswesen zur Förderung der Ressourcenoptimierung umgesetzt werden könnten [11], muss auch die Verteilung von Produktproben berücksichtigt und infrage gestellt werden. Jede einzelne Maßnahme – und besonders solche mit bisher eher gering eingeschätztem Impact – kann also einen signifikanten Einfluss auf die Nachhaltigkeitsstrategie nehmen: Die Umweltauswirkungen von Produktproben beeinträchtigen die Bemühungen des Gesundheitssektors zur Reduktion von Treibhausgasemissionen und erschweren die Erreichung der Klimaneutralität bis 2030.

Gesetzliche Rahmenverordnungen wie die EU-Taxonomie [7], ein Klassifizierungssystem, das Kriterien für die ökologisch nachhaltige wirtschaftliche Aktivität darstellt, und die durch die Europäische Union am 05.01.2023 veröffentlichte Corporate Sustainability Reporting Directive (CSRD) [6], die Unternehmen definierter Größe dazu verpflichtet, Nachhaltigkeitsberichte zu erstatten, werden in naher Zukunft finanzwirtschaftliche Aspekte mit der Einsparung von Treibhausgasemissionen, Ressourcen und Wasserverbrauch verbinden. In diesem Kontext sind Samplings nicht nur ökologisch problematisch, sondern könnten langfristig über das EU-Taxonomie- und CSRD-Reporting das Rating des Unternehmens auf den Finanzmärkten negativ beeinflussen. In einem zunehmend umweltbewussten Marktumfeld drohen auch Imageschäden. Statt Sampling sollten fortschrittliche, messbare und nachhaltige Marketingmaßnahmen bevorzugt werden, die innovative und präzise Zielgruppenansprachen unter Reduktion von Kosten‑/Ressourceneinsatz und Minimierung des eigenen CO2eq-Unternehmensfußabdruckes ermöglichen [4].

## Fazit für die Praxis

- Die Verpackungen dermatologischer Samplings verursachen hohe CO2eq (Treibhausgasäquivalente) und erheblichen Nutzwasserverbrauch.
- Ein Recycling ist abhängig von der Produktgröße und bei Verbundstoffen oft nicht möglich, kleine Verpackungen haben in Bezug auf das Produkt den größten ökologischen Fußabdruck.
- Produktproben erfordern den Einsatz zeitlicher und personeller Ressourcen in Praxen, Kliniken und Apotheken. Ihre Annahme, Sortierung und Entsorgung generieren Kosten. So gefährdet fortgesetztes Sampling das Ziel der Klimaneutralität des Gesundheitswesens bis zum Jahr 2030.
- Sampling ist ein Marketinginstrument mit schlecht messbarem Effekt; die Umstellung auf innovative Marketingstrategien bietet Herstellern die Chance auf Ressourcen- und Kostenreduktion unter Berücksichtigung der EU-Regulatorien.
